# Scaling theory of rubber sliding friction

**DOI:** 10.1038/s41598-021-97921-0

**Published:** 2021-09-15

**Authors:** Reinhard Hentschke, Jan Plagge

**Affiliations:** School of Mathematics and Natural Sciences, University of Wuppertal, Wuppertal, 42097 Germany

**Keywords:** Materials science, Soft materials, Polymers

## Abstract

Current theoretical descriptions of rubber or elastomer friction are complex—usually due to extensive mathematical detail describing the topography of the solid surface. In addition, the viscoelastic properties of the elastomer material itself, in particular if the rubber is highly filled, further increase the complexity. On the other hand, experimental coefficients of sliding friction plotted versus sliding speed, temperature or other parameters do not contain much structure, which suggests that a less detailed approach is possible. Here we investigate the coefficient of sliding friction on dry surfaces via scaling and dimensional analysis. We propose that adhesion promotes viscoelastic dissipation by increasing the deformation amplitude at relevant length scales. Finally, a comparatively simple expression for the coefficient of friction is obtained, which allows an intuitive understanding of the underlying physics and fits experimental data for various speeds, temperatures, and pressures.

## Introduction

The friction of rubber on solid surfaces is technologically important and theoretically challenging. Many applications are related to mobility and transport and consequently the study of rubber friction has a long history (e.g.^1^). Early systematic experimental studies of the frictional behaviour of rubber on solid surfaces, focussing on parameters like sliding speed, temperature, load or surface type, began to appear in the early 1940s and have continued ever since (e.g.^2–9^). Perhaps the most extensive early collection of theoretical ideas, which still can be read with profit today, is a report by Kummer^10^. He already discusses in detail the two mechanisms which are considered to contribute to the friction of rubber: (a) the adhesive contact of interfacial layers depending on the surface free energy of the rubber and the solid surface and (b) energy dissipation caused by the time dependent deformation of the viscoelastic rubber by the asperities of the solid’s surface. About 20 years ago a number of groups began to develop rather sophisticated models for rubber friction^11–14^. A particular element of these theories is the description of surface roughness using the concept of self-similarity and scale invariance, which subsequently has also been pursued by others^15–17^. Especially the theory of Persson^13,14^ et al. underwent substantial extensions over the past 20 years. Nevertheless, the significant role of adhesion is still debated and under active research. While Grosch originally attributed it to molecular detachment of single polymer chains^4^, more recent works favour peeling effects on the continuum level^12,18^ or even meniscus formation of rubber domains without crosslinks^19^. Genovese et al. concluded that even the most elaborate theories contain a significant number of unknown empirical parameters, especially when adhesive effects are included^20^. An instructive, albeit older review, of these theoretical ideas and their practical implications is Ref.^21^. Finally it is worth mentioning that large scale computer simulations have evolved into a valuable tool to study contact interfaces, e.g. using continuum mechanical methods^22^, and the role of the molecular scale in the context of friction, e.g. via molecular dynamics simulations^23^.

The aforementioned key elements of a theory for rubber friction, i.e. adhesion, dissipation in the viscoelastic rubber due to the interaction with the surface’s roughness, as well as the mathematical modelling of the roughness itself, each are complex—even under dry conditions. On the other hand, the measured data for the friction coefficient vs. sliding speed, for instance, feature comparatively little structure—even though the data usually span many decades. It therefore appears reasonable to look for a simpler and more transparent theoretical expression, which still includes all the important parameters, e.g. the interface tension, the loss modulus of the slider, the load, etc. With this in mind, we develop a scaling description of rubber friction on solid surfaces under dry (or almost dry) conditions. Adhesion and hysteresis friction do not enter through separate terms as in other theories. Both are combined from the beginning into a single expression. Energy dissipation caused by the disruption of interface patches during sliding is described via a contribution to the strain amplitude in the interface. The latter couples to the loss modulus via sliding speed induced excitation frequencies. The increase in amplitude drives ’hysteresis friction’ and both, the adhesion and the hysteresis contribution, do depend on the surface roughness of the solid. The particular appeal of the final result for the coefficient of sliding friction is that it combines the key ingredients into a single compact expression permitting an easy understanding of their interdependence. We apply our theory to recent measurements for a racing tire compound, for which we find quite good agreement despite the unavoidable neglect of detail inherent to a scaling approach.

## Scaling theory of sliding friction of elastomers on (almost) dry solid surfaces

The (sliding) friction coefficient $\mu _f$ is defined as

1 $$\begin{aligned} \mu _f = \frac{F_{d}}{F_n}\;. \end{aligned}$$

$F_n$ is the total normal force acting uniformly across a macroscopic area *A* between the sliding body and the surface. $F_{d}$ is the force, which, if multiplied by the macroscopic distance traversed by the sliding body, yields the dissipated energy due to friction between the body and the surface. We re-express $F_n$ as $F_n=P A$ and $F_d$ as $F_d=w_d A$, making momentarily no difference between *A* in the numerator and *A* in the denominator, i.e.

2 $$\begin{aligned} \mu _f = \frac{w_d}{P} . \end{aligned}$$

The quantity $w_d$ is the (rubber) volume density of the dissipated energy given by

3 $$\begin{aligned} w_d \sim E^{\prime \prime } u^2 , \end{aligned}$$

where $E^{\prime \prime }$ is the loss modulus and *u* is the strain amplitude of the rubber in the interface. One might object that this expression is based on a linear relation between stress and strain, which certainly is not true for the important class of highly filled elastomers. But since the standard analysis of stress–strain-curves, even in the case of non-linear materials, is often based on the above relation for $w_d$, it can be regarded as a ’defining’ equation for $E^{\prime \prime }$. Thus

4 $$\begin{aligned} \mu _f \sim \frac{E^{\prime \prime } u^2}{P}\;. \end{aligned}$$

It is worth noting that $E^{\prime \prime } (f)$, where *f* is the frequency of deformation, can be (and usually is) obtained via standard methods and therefore is more readily available than $\mu _f$.

We concentrate first on the low frequency limit, i.e. the sliding speed $v \sim 0$. In this quasi-static limit we define a lateral length (scale) $\lambda _0$ over which a certain surface roughness $h_0$, i.e. $h_0$ is the square root of the mean-square height along the surface or $h_0 \equiv \sqrt{\langle h^2(0) \rangle }$, is sampled. Aside from $\lambda _0$ and its attendant $h_0$ we consider the interface tension $\Delta \gamma$ and the pressure *P*. These quantities are linked via the condition

5 $$\begin{aligned} \frac{1}{2} E \left( \frac{h_0}{\lambda _0} \right) ^2 \lambda _0^3 \sim \Delta \gamma \lambda _0^2 + \frac{1}{2} E \left( \frac{P}{E}\right) ^2 \lambda _0^3\;. \end{aligned}$$

The term on the left is the elastic free energy of a rubber with Young’s modulus *E* within a volume element $\lambda _0^3$. Note that $u\sim h_0/\lambda _0$ is taken to be the average strain in the volume element. The first term on the right is the reversible surface work during the formation of a rubber-solid contact area of size $\lambda _0^2$. Finally, the second term on the right is the work done by the pressure $P \sim E \varepsilon$, where $\varepsilon$ is the strain within the volume element due to the pressure. Hence we obtain the equilibrium strain (amplitude)

6 $$\begin{aligned} u_{eq} = \left( \frac{2 \Delta \gamma }{E \lambda _0} + \frac{P^2}{E^2}\right) ^{1/2} \end{aligned}$$

and using Eq. (5) we can express $\lambda _0$ in terms of the other quantities, i.e.

7 $$\begin{aligned} \lambda _0 \sim \frac{E }{ P^2} \left( \sqrt{\Delta \gamma ^2 + h_0^2 P^2} - \Delta \gamma \right) = \left\{ \begin{array}{cc} \frac{E h_0^2}{2 \Delta \gamma } &{} P \rightarrow 0 \\ \frac{E h_0}{P}&{} \Delta \gamma \rightarrow 0 \end{array} \right. \,. \end{aligned}$$

When the sliding speed increases we expect that the actual amplitude becomes less than $u_{eq}$. We can also express *u* in terms of a new (dynamic) characteristic length $\lambda$ (generally different from $\lambda _0$) via

8 $$\begin{aligned} u \sim h/\lambda \;. \end{aligned}$$

Here the roughness *h* is defined analogous to $h_0$ but on the lateral length scale $\lambda$ instead of $\lambda _0$. The relation $h \sim \lambda ^{H}$^21^, where *H* is the Hurst exponent of the solid surface, can be used to eliminate *h* from the previous equation, i.e.

9 $$\begin{aligned} u \sim \frac{h_0}{\lambda _0} \left( \frac{\lambda }{\lambda _0} \right) ^{H-1}\;. \end{aligned}$$

Note that in the limit $\lambda =\lambda _0$ this means that $u \sim h_0/\lambda _0=u_{eq}$, corresponding to the sliding speed $v=0$. We therefore connect $\lambda$ to the sliding speed via

10 $$\begin{aligned} \lambda -\lambda _0 = \tau v\;, \end{aligned}$$

where $\tau$ is a characteristic time. This also means that $\lambda$ increases with *v*. The implication of this is discussed below. Hence

11 $$\begin{aligned} u \sim u_{eq} \left( 1+ \frac{ \tau v}{\lambda _0}\right) ^{H-1}\;. \end{aligned}$$

Linking the slider’s velocity *v* to the excitation frequency *f* can be accomplished as follows. The rate at which the surface is sampled laterally is $v/\lambda _0$. Here we use $\lambda _0$ as a natural length instead of $\lambda$, because according to (10) $v/\lambda$ is independent of *v* for $\lambda \gg \lambda _0$. We expect that the excitation rate, or frequency *f*(*v*), experienced by the slider, should scale according to a power law, i.e. $f \sim (v/\lambda _0)^\alpha$. In the simplest case we may expect $\alpha =1$. However, motivated by $h \sim \lambda ^H$, which means that the sampling of a lateral distance $\lambda$ implies the sampling of an interface width on the order of $\lambda ^H$, we assume the existence of an analogous scaling tying the lateral rate $v/\lambda _0$ to the roughness induced excitation rate *f*(*v*). This then implies $\alpha =H$. Hence

12 $$\begin{aligned} \frac{f(v)}{f_{0,\perp }} = \left( \frac{v/{\lambda _0}}{f_{0,\parallel }} \right) ^H\;. \end{aligned}$$

The quantities $f_{0,\perp }$ and $f_{0,\parallel }$ are suitable frequency units of *f* and $v/{\lambda _0}$ respectively. It is natural to set $f_{0,\perp }=f_{0,\parallel }=1$ Hz. Since the entire argument also holds if we replace $\lambda _0$ by a constant times $\lambda _0$, we arrive at

13 $$\begin{aligned} f(v) = f_{0,\perp } \left( \frac{v/{(z \,\lambda _0)}}{f_{0,\parallel }} \right) ^H\;, \end{aligned}$$

where *z* is a dimensionless constant of order unity.

Putting everything together we obtain for the coefficient of sliding friction

14 $$\begin{aligned} \mu _f = \mu _{f,c} \frac{E^{\prime \prime }( f(v)) u_{eq}^2}{P} \left( 1+ \frac{\tau v}{\lambda _0}\right) ^{2(H-1)}. \end{aligned}$$

Here *f*(*v*) and $u_{eq}$ are given by Eqs. (13) and (6), respectively, and $\lambda _0$ is given by Eq. (7). The factor $\mu _{f,c}$ is an adjustable constant. The quantity $h_0$ and the characteristic time $\tau$ are, for the moment, adjustable parameters as well. *H* can be obtained from suitable measurements of the surface topography and usually ranges between 0.5 and 1 for engineering surfaces. The interface tension $\Delta \gamma$ is a measurable quantity as well. So is the modulus *E* in the limit of low frequencies - even though it exhibits a strong dependence on strain amplitude in the case of a highly filled elastomers (Payne effect (cf. for instance Ref.^24^)). In addition, the temperature dependence of the Payne effect is quite different from that of an unfilled elastomer. In the latter case we can use time-temperature superposition to convert temperature into an attendant frequency. This is accomplished most conveniently using the Williams-Landel-Ferry (WLF)-description of the shift factor $a_T$, i.e.

15 $$\begin{aligned} \log _{10}(a_T) =-c_1 (T-T_{\text{ ref }})/(c_2 + (T-T_{\text{ ref }}))\, \end{aligned}$$

where $T_{\text{ ref }}$ is a reference temperature and $c_1$ and $c_2$ are constants. The frequency $E^{\prime \prime } (f(T))$ is then obtained from $E^{\prime \prime } (f(T_{\text{ ref }}))$ via $f(T) = a_T \, f(T_{\text{ ref }})$. If the temperature dependence of the aforementioned Payne effect does not enter too strongly into the calculation of $\mu _f$, then its temperature dependence can be described by $a_T$. We will see later that the relevant length scales induce excitation frequencies near the glass transition, where the modulus is dominated by the polymer matrix.

## Comparison to experimental data

**Figure 1:**
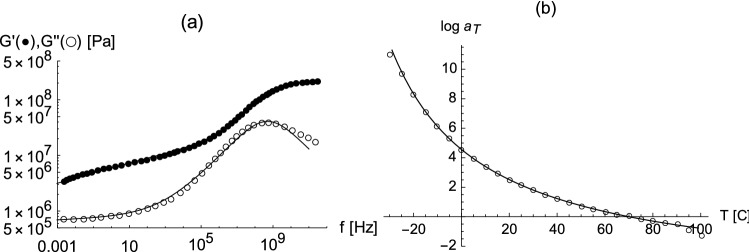
(**a**) Shear storage modulus $G^{\prime } = E^{\prime }/3$ (solid circles) and shear loss modulus $G^{\prime \prime } = E^{\prime \prime }/3$ (open circles) vs. frequency *f*. The symbols are taken from the respective master curves in Fig. 8d of Ref.^25^ obtained at a reference temperature of 70 °C and with a shear strain amplitude of 0.2% . The solid lines are drawn using Eq. (16) with $E_1^{\prime }=600$ MPa, $x_1^{\prime }= 0.25$, $E_2^{\prime }=150$ MPa, $x_2^{\prime }= 0.05$ and $E_1^{\prime \prime }=240$ MPa, $x_1^{\prime \prime }= 0.35$, $E_2^{\prime \prime }=5.1$ MPa, $x_2^{\prime \prime }= 0.025$ and $t_g=2 \cdot 10^{-9}$ s. (**b**) $\log _{10}a_T$ vs. temperature *T*. The symbols are taken from Fig. 7 of Ref.^25^. Here the solid line is a WLF fit with $c_1=-4.41$, $c_2=137,64$ ${}^o$C and $T_{\text{ ref }}=70$ ${}^o$C.

In the following we concentrate on data in Ref.^25^ (based on the thesis work in Ref.^26^) for a racing tire tread compound. The symbols in Fig. 1 represent a master curve for the loss modulus $E^{\prime \prime }(f)$ of this slider material obtained at a reference temperature of 70 ${}^o$C. In the following it will be convenient to use the two-term empirical fit functions

16 $$\begin{aligned} E^{\prime }(f) = \sum _{i=1}^2 E_i^\prime \frac{(t_g f)^{2 x_i^\prime }}{(t_g f)^{2 x_i^\prime } +1} \;, \qquad E^{\prime \prime }(f) = \sum _{i=1}^2 E_i^{\prime \prime } \frac{(t_g f)^{x_i^{\prime \prime }}}{(t_g f)^{2 x_i^{\prime \prime }} +1} \;, \end{aligned}$$

which essentially is a generalization of the Maxwell model, included in Fig. 1a. Here the quantities $t_g$, $E_i^\prime$, $E_i^{\prime \prime }$, $x_i^\prime$, and $x_i^{\prime \prime }$ are fit parameters. Panel (b) in this figure shows the shift factor $a_T$ obtained in the above reference. Note that our representations of $E^\prime$ and $E^{\prime \prime }$ are to be regarded as fit functions which do not necessarily fulfill the Kramers-Kronig relations. It will also be convenient to use the WLF function (15) for $a_T$ instead of the data points themselves.

**Figure 2:**
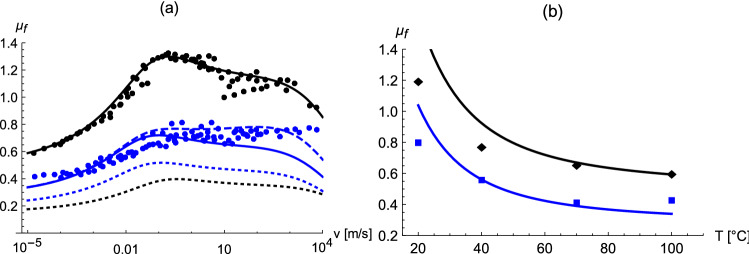
(**a**) Master curves from Fig. 10 of Ref.^25^ for coarse granite. Black dots: $P=1$ bar; blue dots: $P=7$ bar. Reference temperature: $T=70$ °C. The lines represent our scaling theory. Solid black line: Fit of $\mu _f(P=1)$ using Eq. (14); solid blue line: $\mu _f (P=7)$ using Eq. (14) with the fit parameter values previously obtained for $\mu _f (P=1)$; dashed blue line: same as $\mu _f (P=7)$ but with $H=0.868$ instead of $H=0.858$; the dotted lines correspond to the solid lines of the same colour when $\Delta \gamma =25$ mJ/m^2^ instead of 50 mJ/m^2^. (**b**) Experimental dependence of $\mu _f$ on temperature *T* according to Fig. 9 in Ref.^25^ (first data point along the velocity axis). The sliding speed is $10^{-4}$ m/s. Black dots: $P=1$ bar; blue dots: $P=7$ bar. The line shows the theoretical result obtained with unaltered parameter values.

Figure 2a shows experimental master curves for $\mu _f$ at $P=1$ bar and $P=7$ bar, respectively, from Fig. 10 of Ref.^25^ for coarse granite. Note that the reference temperature, i.e. 70 °C, is the same as in Fig. 1. The lines in the figure are calculated via Eq. (14). Here $\mu _{f,c}=2.48$, $h_0 = 2.1 \times 10^{-7}$ m, $\tau = 2.0 \times 10^{-5}$ s, $z=2.3$, and $H=0.858$. *z* is not a very sensitive parameter. Choosing the present value instead of simply one, yields a slight improvement of the fit to the data for 1 bar for sliding speeds between $10^{-5}$ to $10^{-2}$ m/s. It is worth noting that the above assertion $\alpha =H$ performs greatly superior compared to $\alpha =1$. In Fig. 6 of Ref.^25^ Lang and Klüppel obtain $H \approx 0.65$ on coarse granite. The authors of Ref.^21^ find that *H* on granite, i.e in principle the same solid as in Ref.^25^, is very close to unity (cf. Fig. 11 in the reference). Our current values for *H* are bracketed by these findings. It is worth remarking that *H* is measured for the solid surface alone. Here, however, we consider an interface between two surfaces, each characterized by its own roughness, which may very well affect the proper exponent value. In addition we use $E = 9 \times 10^{6}$ Pa, in accordance with the low frequency value of the material’s storage modulus shown in Fig. 7 of Ref.^25^. For the interface tension we assume $\Delta \gamma =50$ mJ/m^2^. Even though this is a guess, similar numbers have been measured for not too different systems^26,27^. Note that $\lambda _0\approx 1.3 \cdot 10^{-6}$ m and $u_{eq} \approx 17$ % when $P=1$ bar and $\lambda _0\approx 0.6 \cdot 10^{-6}$ m and $u_{eq} \approx 33$ % when $P=7$ bar. Note also that the parameters are adjusted to yield the best fit to the data at $P=1$ bar. The theoretical curve for $P=7$ bar (solid blue line in Fig. 2a) is obtained with the same parameter values without additional adjustment. The dashed blue curve is obtained with $H=0.868$ instead of $H=0.858$. The motivation for including this curve as a comparison is the idea that increasing the pressure conceivably affects the interface and thus the value of the scaling exponent. The interplay between pressure and interface tension is demonstrated by the dotted lines in Fig. 2a, corresponding to the solid lines of the same colour when $\Delta \gamma =25$ mJ/m^2^ instead of 50 mJ/m^2^. Note that the significant reduction of $\Delta \gamma$ not only reduces $\mu _f$, as expected, but also has a stronger effect at $P=1$ bar compared to $P=7$ bar. $\Delta \gamma$ usually is varied by liquid films (e.g. water/soap-mixtures) wetting the solid surface. However, the current theory is for dry surfaces only (and perhaps remains applicable for ’frozen’ films whose thickness is a few molecular diameters), which means that data obtained on liquid films cannot be used for direct comparison.

**Figure 3:**
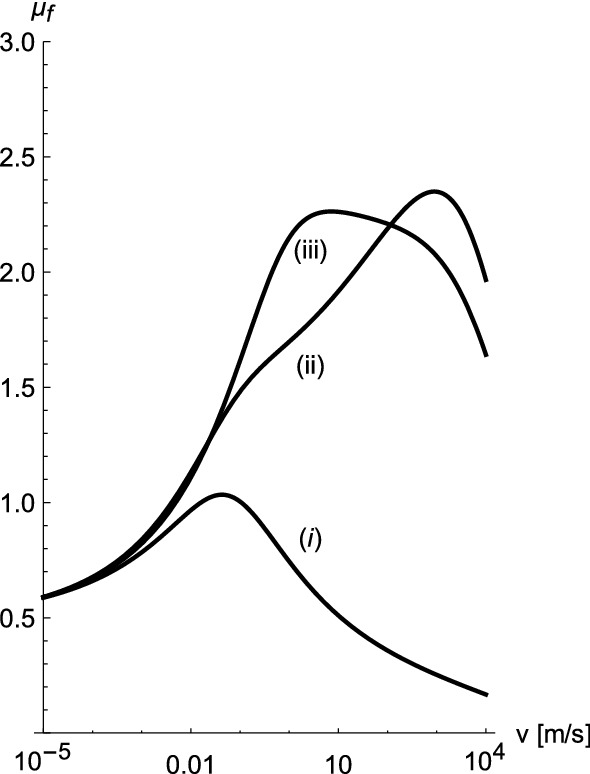
Variation of the friction coefficient $\mu _f$ vs. sliding speed *v* relative to the theoretical curve in the previous figure for $P=1$ bar. (i) $H=0.8$ (ii) $H=0.89$ (iii) previous $\tau$ times 1/10.

**Figure 4:**
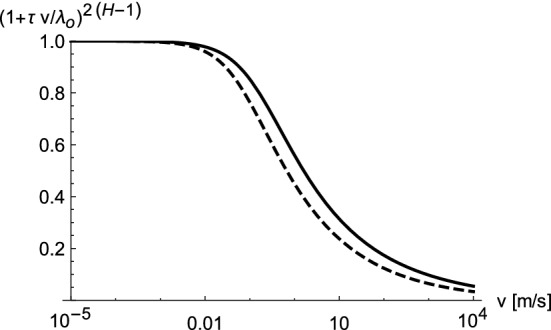
The effect of $\tau (f)$ (solid line) compared to $\tau \equiv \tau _c=2 \cdot 10^{-5}$ s used to obtain the above results (dashed line). All remaining parameters are identical to those of the $P=1$ bar result shown in Fig. 2a.

Generally, the position of the first peak of $\mu _{f}$ is controlled by the value of $\tau$, i.e. the characteristic time which indicates the departure of the strain amplitude from its equilibrium value. The position of the shoulder or, depending on parameter values, second peak (’hysteresis peak’) beyond which $\mu _f$ strongly decreases, on the other hand, is determined by the peak of $G^{\prime \prime }(f)$. The shape of $\mu _f$ vs. $\log (v)$, i.e. a peak at small *v* and a shoulder at large *v* or a mere shoulder at small *v* and a pronounced peak at large *v* or just a rounded single peak (cf. for instance Figs. 4, 5, and 7–10 in Ref.^4^), largely depends on the relative magnitude of $\tau$ compared to the Hurst exponent *H*. Increasing $\tau$ will result in a smaller first peak, which will become a mere shoulder of the ’hysteresis peak’ if *H* is increased. Example variations are depicted in Fig. 3. The parameters are identical to those used to calculate the (solid) theoretical curve in Fig. 2 for $P=1$ bar. Only a single parameter, indicated in the figure caption, is changed for each curve. Notice that *H* has little influence in the limit of small sliding velocity but a pronounced effect otherwise. Decreasing $\Delta \gamma$ also decreases $\mu _f$ - as mentioned above. Likewise, increasing *E*, i.e. increasing the sliders ’hardness’, will also decrease $\mu _f$, in accord with the experimental observations. Finally, panel (b) in Fig. 2 shows the temperature dependence of $\mu _f$ at slow sliding speeds. The solid lines are results of this theory using the same parameter values as in the previous figure. The temperature dependence is introduced into Eq. (14) using the shift factor (15) as explained above.

## Discussion and conclusion

The present scaling theory of sliding friction yields a simple expression, i.e. Eq. (14), which nevertheless yields good accord with experimental measurements. The slider material enters via its loss modulus $E^{\prime \prime }$, its modulus in the low frequency limit *E* and its shift factor $a_T$. The topography of the surface material on the other hand enters via the Hurst exponent *H*. The chemical nature of the two materials in the contact area enters via the interface tension $\Delta \gamma$. Load, as an external parameters, enters in terms of pressure *P*. Another interface parameter is the characteristic time $\tau$, defined in Eq. (10), which governs the crossover from sliding speeds at which the strain amplitude is essentially given by $u_{eq}$ to faster sliding speeds when this is no longer the case. Due to its relative simplicity, the theory allows easy access to the interplay between the aforementioned quantities, which is useful during the development of new rubber compounds—especially in the tire industry.

It is important to note that the scaling relation $h \sim \lambda ^{H}$, used to obtain (9), holds only when $\lambda < \lambda _{\text{ max }}$, i.e. at $\lambda \approx \lambda _{\text{ max }}$ the surface roughness reaches its limiting value $\sigma$ and remains constant for larger $\lambda$. Hence for $v> v_{\text{ max }}=(\lambda _{\text{ max }} - \lambda _0)/\tau$ relation (9) can no longer be used. It can be replaced by

17 $$\begin{aligned} u \sim \frac{\sigma }{\lambda } \sim \frac{\sigma }{\lambda _0 + \tau v} \;. \end{aligned}$$

Using $\sigma / h_0 \sim (\lambda _{\text{ max }} / \lambda _0)^H \sim (1+ \tau v_{\text{ max }}/\lambda _0)^H$ this can be rewritten as

18 $$\begin{aligned} u \sim u_{eq} \frac{(1+ \tau v_{\text{ max }}/\lambda _0)^H}{1 + \tau v/\lambda _0} \qquad (v>v_{\text{ max }})\;. \end{aligned}$$

Note that (18) agrees with (11) at $v=v_{\text{ max }}$. However, the resulting steeper decrease of *u*(*v*) with increasing sliding velocity does not manifest itself in the data considered here and we have not included this additional crossover-behaviour into the expression for $\mu _f$. In principle $v_{\text{ max }}$ can be estimated via Eq. (10) which links $\lambda$ to *v*. The approximate breakdown of $h \sim \lambda ^{H}$ for granite is depicted in Fig. 6 of Ref.^25^ ($\lambda > 3 \cdot 10^{-3}$ m or $v_{\text{ max }}\ge 100$ m/s using the above value for $\tau$) as well as in Fig. 11 of Ref.^21^ (here the scaling holds even at values of $\lambda$ corresponding to $v_{\text{ max }} \approx 1000$ m/s). In the former case we should see a steeper decrease but not in the latter, i.e currently the point is undecided.

In Fig. 1 we have included the storage modulus vs. frequency, which increases by about a factor of 60 over the frequency range considered here. Therefore the question arises why Eq. (14) contains only $E=E^\prime (f\rightarrow 0)$. Note that *E* is introduced in Eq. (5), which is based on the low frequency limit, determining $u_{eq}$. When the frequency rises due to the increasing velocity it is only relation (8) which is valid. This relation contains no particular reference to the slider’s material. In Eq. (9), obtained from (8) via $h \sim \lambda ^H$, $u_{eq}$ enters because *h* is expressed in units of $h_0$ and $\lambda$ in units of $\lambda _0$. Thus, the dynamic elasticity of the slider does not enter via *E*. It enters via the characteristic time $\tau$ in Eq. (11), which describes the reduction of the strain amplitude when the sliding speed is increased. Nevertheless, the above results are obtained using a $\tau$ independent of $E^\prime (f)$. A possible dependence of $\tau$ on $E^\prime$ is $\tau \sim (E^\prime )^{-1/2}$. This is motivated by the proportionality of the transverse velocity of sound in an isotropic elastic medium to the square root of the Young’s modulus. In other words, the time for a transverse wave to traverse a fixed distance is proportional to the inverse of the square root of the Young’s modulus. Thus, we may express $\tau (f)$ via $\tau (f) =\tau _c (E/E^\prime (f))^{1/2}$. Here $\tau _c$ is our constant $\tau$ used thus far, i.e. in the low frequency limit $\tau (f)$ and $\tau _c$ coincide, whereas at high frequencies $\tau (f) <\tau _c$. Figure 4 compares the factor $(1+ \tau v/\lambda _0)^{2 (H-1)}$ (cf. Eq. (14)) with a variable $\tau =\tau (f)$, where we use the fitted $E^\prime$ in (16) and the constant $\tau = \tau _c=2 \cdot 10^{-5}$ s used in all previous figures. As before we connect *f* with *v* via Eq. (13). The strong variation of $E^\prime$ translates into a rather small variation of $\mu _f$. Thus, in the examples considered here it is permissible to use a constant $\tau$. Nevertheless, if we use $\tau (f)$ instead of the above constant value, then we must change $\tau _c$ from $2 \times 10^{-5}$ s to $3 \times 10^{-5}$ and $H=0.858$ to $H=0.845$ in order to obtain theoretical curves identical in quality to those in Fig. 2.
